# Partial Depletion of Peripheral M1 Macrophages Reverses Motor Deficits in MPTP-Treated Mouse by Suppressing Neuroinflammation and Dopaminergic Neurodegeneration

**DOI:** 10.3389/fnagi.2018.00160

**Published:** 2018-06-05

**Authors:** Aijuan Yan, Yu Zhang, Jingya Lin, Lu Song, Xijin Wang, Zhenguo Liu

**Affiliations:** Department of Neurology, Xinhua Hospital Affiliated to Shanghai Jiao Tong University School of Medicine, Shanghai, China

**Keywords:** M1 macrophages, T cells, neuroinflammation, microglia, Parkinson's disease

## Abstract

**Background:** Neuroinflammation plays an important role in the pathogenesis of Parkinson's disease (PD). Inflammatory cytokines in the peripheral immune system can induce neuroinflammation in central nervous system (CNS). Whether the peripheral immune system is involved in PD is unclear. The present study investigated the contribution of the peripheral immune system to the neuronal loss in the 1-methyl-4-phenyl-1,2,3,6-tetrahydropyridine(MPTP) model of PD.

**Methods:** MPTP was intraperitoneally injected into mice to generate a PD model. Mice received clodronate liposomes every 3 days to deplete peripheral macrophages. The percentages of macrophages were measured by flow cytometry at 1, 3, and 7 days after MPTP injection. Neurobehavioral parameters, protein expression, inflammatory cytokines release, and microglia activation were measured by the open field test, western blotting, quantitative polymerase chain reaction (qPCR), and immunofluorescence staining, respectively at 7 days after MPTP injection.

**Results:** Our study revealed that intraperitoneal injection of MPTP could increase peripheral M1 macrophages levels. It also can induce T cells infiltration and cytokine release. Depletion of M1 macrophages by clodronate liposomes suppressed these inflammatory effects and blunted the loss of TH+ nigral neurons and functional impairments caused by MPTP.

**Conclusion:** Our results indicated the critical role of M1 macrophages in the pathogenesis of PD and proposed inhibition of M1 macrophages as a promising therapeutic approach for neurodegeneration.

## Introduction

Parkinson's disease (PD) is one of the most common neurodegenerative diseases and is characterized by the progressive death of dopaminergic neurons in the substantia nigra pars compacta (SNpc; Ferrari and Tarelli, 2011). Neuroinflammation is a key factor in the degeneration of neurons in the SNpc throughout the initiation and progression of PD (Appel, 2012; Chen et al., 2016). Several clinical studies have reported on the association of alterations in the peripheral immune response associated with familial PD to neuroinflammation and neurodegeneration (Mutez et al., 2011; Dzamko et al., 2016). However, the function of the peripheral immune system in PD is not fully understood.

Macrophages are the pivotal regulatory cells of the peripheral immune system (Edholm et al., 2017). One of the critical characteristics of macrophages is the ability to adopt a variety of different activation states in response to the toxic environment they encounter. Differentiated macrophages are referred to classically activated M1 macrophages or alternatively activated M2 macrophages depending on their activation state. However, this kind of classification is now considered an oversimplified approach that does not adequately describe the spectrum of macrophage. For instance, the identification of tumor associated macrophages (TAMs), which do not fit nicely into the criteria for M1 or M2 macrophages complicates this system (Chinetti-Gbaguidi et al., 2015). M1 macrophages release pro-inflammatory factors and chemokines such as interleukin-1β (IL-1β), tumor necrosis factor-α (TNF-α), and monocyte chemotactic protein-1 (MCP-1), as well as oxidative metabolites such as iNOS and superoxide to recruit additional inflammatory cells (Barrientos et al., 2008; Yan et al., 2016), express high levels major histocompatibility complex II (MHC II) molecules. M1 macrophages are more efficient than M2 macrophages at presenting antigens and have more capacity to prime naive T cells. In contrast, M2 macrophages produce anti-inflammatory cytokines including interleukin-10 (IL-10), and they also promote tissue remodeling (Locati et al., 2013; Murray et al., 2014). M2 macrophages express low levels of MHC II. Differential abundance of M1 and M2 macrophages has also been found associated with different disease phenotypes (Wang et al., 2011). As the disease continues to progress, M2 macrophages are gradually replaced by M1 macrophages (Gordon and Martinez, 2010). This indicates that the inhibition of M1 macrophages and the promotion of M2 macrophages could alleviate inflammatory response. It would be meaningful to investigate whether the macrophages play an important role in the pathogenesis of PD.

To directly explore the role of periphery macrophages in the pathogenesis of PD, we depleted the macrophages by intraperitoneally injecting clodronate liposomes, which has been used in several stress and LPS studies (Kotter et al., 2001; Zhu et al., 2015; Reiling et al., 2018). We used the 1-methyl-4-phenyl-1,2,3,6-tetrahydropyridine (MPTP) induced mouse model of PD to assess the activation status of macrophages in peripheral immune system. Then, we mainly explored the role of partial depletion of M1 macrophages by clodronate liposomes in neuroinflammation and dopaminergic neurodegeneration. As well, we studied the effect of clodronate liposomes on functional impairments caused by MPTP in the PD model. Our research may give a new clue for the implication of peripheral immune system in PD pathogenesis.

## Materials and methods

### Animals

Male C57BL/6 mice were purchased from Shanghai SLAG Laboratory Animal Corporation (Shanghai, China). The mice (10–12 weeks old, weighing 25–30 g) were used for this study. The mice were kept in a temperature-controlled room (22 ± 1°C) at 50–60% relative humidity, with a 12/12-h light–dark cycle, and had free access to food and water. This study was carried out according to the guidelines of the National Institutes of Health (publication No. 80-23). All procedures were approved by the Institutional Review Board of Xinhua Hospital affiliated to Shanghai Jiao Tong University Medical School.

### Drugs and treatments

**Figure 1:**
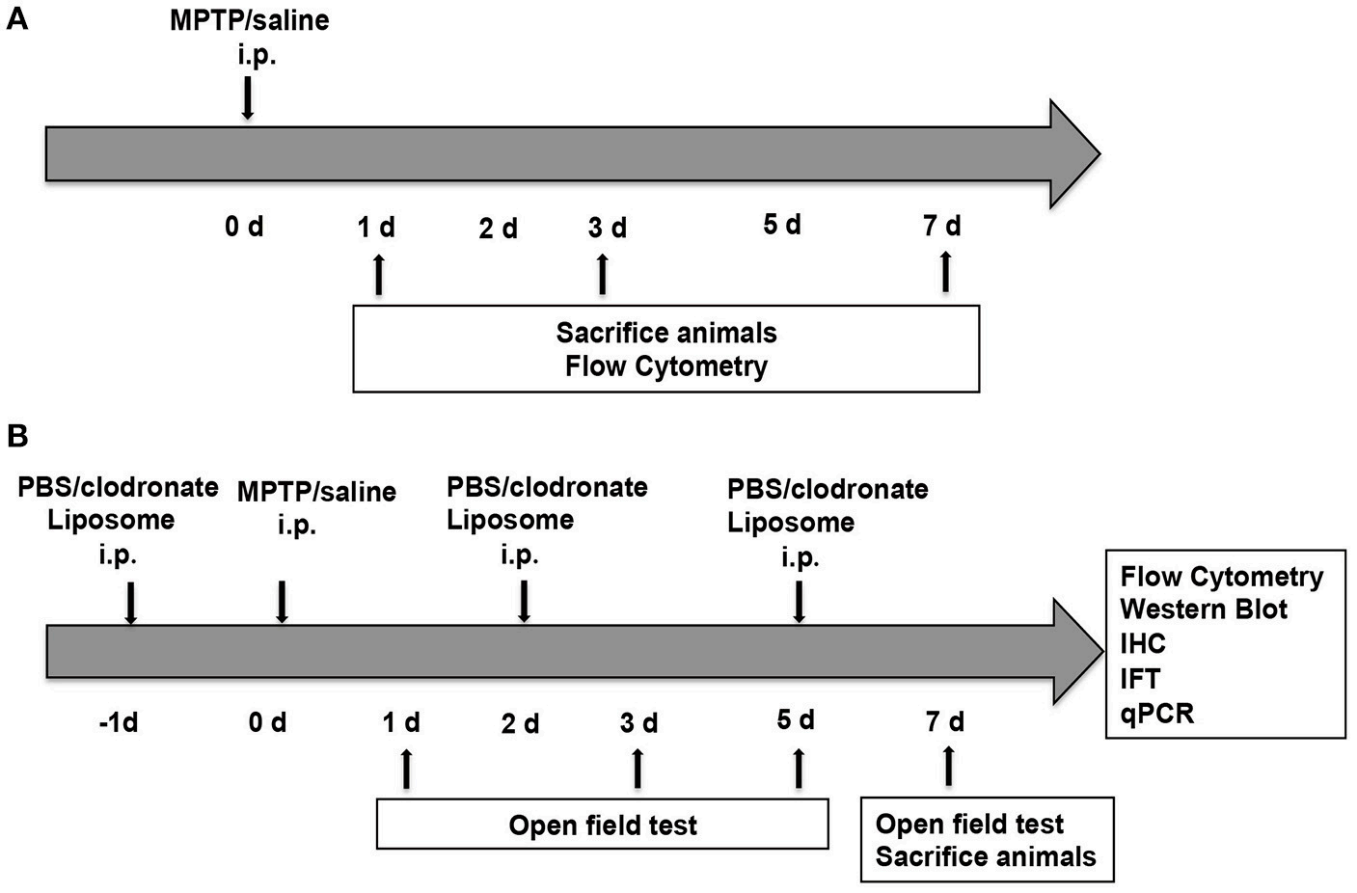
Experimental design. **(A)** Mice (*n* = 8) were administered four intraperitoneal (i.p.) injections of MPTP (20 mg/kg) or 0.9% saline at 2 h intervals. The mice were sacrificed at 1, 3, and 7 days after the first MPTP injection for flow cytometry. **(B)** Mice (*n* = 32) were administered four i.p. injections of MPTP (20 mg/kg) or 0.9% saline at 2-h intervals in addition to PBS liposomes (200 μL prepared) or clodronate liposomes (200 μL prepared) at 1 day before and 2 and 5 days after MPTP injection. Mice were divided into four groups, namely, the PBS lipo-saline group (*n* = 8), the clodronate lipo-saline group (*n* = 8), the PBS lipo-MPTP group (*n* = 8), and the clodronate lipo-MPTP group (*n* = 8). Mice were sacrificed 7 days after the first MPTP injection for flow cytometry, western blotting, RT-PCR and immunofluorescence.

Two groups of C57BL/6 mice received four intraperitoneal (i.p.) injections of MPTP (20 mg/kg; Sigma-Aldrich Chemical, MO, USA) or 0.9% saline at 2-h intervals. These mice were sacrificed at 1, 3, and 7 days after the first MPTP injection (Figure 1A). Another two groups of C57BL/6 mice received i.p. injections of PBS liposomes (200 μL of the order preparation; Nico van Rooijen; Amsterdam, Netherlands) or clodronate liposomes (200 μL of the order preparation) every 3 days (Ma et al., 2016). The next day, the experimental mice received four i.p. injections of MPTP at 2-h intervals, whereas the control group received an equivalent volume of 0.9% saline. This strategy ensured persistent depletion of activated macrophages for the full duration of PD (Zhu et al., 2015). These mice were sacrificed at 7 days after the first MPTP injection (Figure 1B).

### Open field test

After 1, 3, 5, or 7 days of intraperitoneal injection of MPTP, motor behavior was analyzed in an open-field test. The open-field test is a useful method for assessing spontaneous locomotor activity in models of PD (Huang et al., 2014). The apparatus consisted of a square arena with a surrounding wall; infrared beam-break sensors were used to detect movement. The square floor was divided by gridlines into 16 small squares. Each mouse was placed into the center of the field, and its behavior was observed. The number of gridline crossings and total distance traveled were measured by an experimenter seated quietly approximately 1.0 meter away. The following parameters were analyzed by the software of SuperMaze V2.0 (shanghai xin ruan, China) over a 5-min period: total distance traveled and number of gridline crossings. We submitted the original video-tracking of mice in the open field test in the Supplementary Videos 1–10.

### Spleen cell isolation and flow cytometry

Single-cell suspensions of spleen tissue were isolated as described previously with some modifications (Yan et al., 2016). Briefly, mice were anesthetized and perfused to remove peripheral blood on days 1, 3, and 7 after MPTP injection. To obtain single-cell suspensions, we isolated the spleens and ground them in PBS by mechanical trituration through a 100-μm cell strainer (Corning, New York, NY). The cell suspension was centrifuged for 5 min at 1,000 rpm at 4°C, and the supernatant was discarded. Red blood cells were removed by resuspending the cells in red blood cell lysis buffer (Beyotime Institute of Biotechnology) for 1 min. Then, the cell suspension was centrifuged again for 5 min at 1,000 rpm at 4°C, and the supernatant was discarded. After being washed in PBS, the remaining cells were resuspended in 300 μl of PBS and stained for 30 min at 4°C with different combinations of FITC rat anti-mouse CD11b (cat.no.557396, 1:200, BD Pharmingen; Groves et al., 2015) and PE rat anti-mouse MHC II (cat.no.557000, 1:200, BD Pharmingen; Wang et al., 2011). Then, the cells were centrifuged for 5 min at 1,000 rpm at 4°C. After the supernatant was discarded, the cells were washed once with PBS, resuspended in 300 μl of PBS, and analyzed by flow cytometry (BD Biosciences). A minimum of 10,000 cells were acquired for each sample. Data were analyzed using FlowJo (FlowJo, Ashland, OR).

### Immunohistochemistry

Immunohistochemical staining of mouse brain sections was performed as previously described (Luster et al., 2005). Briefly, mice were transcardially perfused with ice-cold phosphate-buffered saline (PBS), followed by 4% paraformaldehyde (PFA) (Sigma-Aldrich) in 0.1 M phosphate buffer. The brains were removed, post fixed for 24 h in 4% PFA, and then cryoprotected in a 30% sucrose solution in PBS (pH 7.4). Subsequently, the brains were frozen, and serial coronal sections (cut thickness: 20 μm) were cut with a microtome. The brain sections were incubated with 0.3% H_2_O_2_ for 30 min at room temperature. After the sections were rinsed with PBS, 0.3% Triton X-100 was added for 10 min, after which the coronal sections were incubated with serum for 1 h at room temperature and then incubated with a rabbit polyclonal anti-TH (cat.no.41528, 1:500, Abcam) (Li et al., 2016) or rabbit monoclonal anti-CD3 (cat.no.16669, 1:500, Abcam) (Luo et al., 2018) at 4°C overnight. Staining was carried out by the ABC method (3,3′-diaminobenzidine (DAB) as the peroxidase substrate. TH-positive DA neurons and CD3-positive T cells from the SNpc region were examined under an optical microscope. Stereological counting of TH-positive neurons and CD3-positive T cells conducted using the method described by previous studies (Hunot et al., 2004; Brochard et al., 2009).

For microglia, brain sections (20 μm in thickness) were fixed with 4% PFA for 15 min and then incubated in PBS for 10 min. Slides were blocked for 1 h in 10% normal donkey serum and then incubated in rabbit monoclonal anti-Iba1 (cat.no.178847, 1:200 dilution, Abcam; Batbold et al., 2017) at 4°C overnight. After being washed three times with PBS, brain sections were incubated with the corresponding donkey anti-rabbit secondary antibody conjugated to Alexa Fluor 488 (cat.no.1834802, 1:400 dilution, Life Technologies)(Yan et al., 2016). 4′,6-Diamidino-2-phenylindole (DAPI) (cat.no.c1002, 1:500 dilution, Beyotime Institute of Biotechnology, China) was used to stain the nuclei. The brain sections were viewed using a fluorescence microscope (Leica, Solms, Germany). The mean numbers of Iba1^+^ microglia were counted in areas of interest measuring 150 × 150 μm in three SN and three striatum sections per mouse.

### Quantitative polymerase chain reaction

The mice from each group were sacrificed by anesthetic overdose 7 days after the MPTP injection. The brains were immediately removed, and tissue was collected from the striatum and SN for quantitative real-time PCR analysis. Total RNA from the brain tissue was isolated using TRIzol Reagent (Life Technologies) and was reverse transcribed to cDNA using the PrimeScript RT reagent kit (TaKaRa). qPCR was performed using a SYBR Green kit (TaKaRa) according to the manufacturer's instructions. qPCR was performed using the following conditions: denaturing at 95°C for 10 s, followed by 40 cycles of 95°C for 5 s, and 60°C for 30 s. The data were analyzed by the comparative threshold cycle (Ct) method, and the results were expressed as fold difference normalized to ribosomal phosphoprotein P0 (Rplp0). The primer sequences were as follows: IL-1β (F: GCAACTGTTCCTGAACTCAACT, R: ATCTTTTGGGGTCCGTCAACT); IL-6 (F: TAGTCCTTCCTACCCCAATTTCC, R: TTGGTCCTTAGCCACTCCTTC); TNF-α (F: CCCTCACACTCAGATCATCTTCT, R: GCTACGACGTGGGCTACAG); Rplp0 (F: AGATTCGGGATATGCTGTT GGC, R: TCGGGTCCTAGACCAGTGTTC).

### Western blotting

Protein samples were prepared by homogenizing spleens in standard lysis buffer. The protein concentration was measured using a BCA kit (Thermo Scientific). Proteins were separated by SDS-PAGE and transferred onto a nitrocellulose membrane. The membranes were blocked with 5% non-fat dried milk for 1 h and incubated with rabbit monoclonal anti-iNOS (cat.no.136918, 1:1,000, Abcam; Lizano et al., 2017), rabbit monoclonal anti-Arg-1 (cat.no.93668, 1:1,000, Cell Signaling Technology; Mogami et al., 2017), rabbit monoclonal anti-p-NF-κB (cat.no.3033, 1:1,000, Cell Signaling Technology; Yan et al., 2017), rat monoclonal anti-MHC II (cat.no.139365, 1:1,000, Abcam; Mori et al., 2015) and rabbit monoclonal anti-β-actin (cat.no.4970, 1:1,000, Cell Signaling Technology; Liu et al., 2018) overnight at 4°C. After being washed with Tris-buffered saline with TWEEN 20 (TBST) buffer, the membranes were incubated with anti-rabbit (cat. no. 7074, 1:2,000, Cell Signaling Technology) or anti-rat (cat. no. 7077, 1:2,000, Cell Signaling Technology) horseradish peroxidase-conjugated secondary antibodies for 1 h at room temperature and then subjected to chemiluminescent detection according to the manufacturer's instructions (Millipore).

### Statistical analysis

All statistical analyses were performed using GraphPad Prism v6.0. For comparison between the two groups, statistical significance was determined through Student's *t*-test. For comparison among multiple groups, statistical significance was evaluated using one-way ANOVA followed by a Student-Newman-Keuls test. Data were expressed as the mean ± standard error of the mean (SEM). *p* < 0.05 were considered statistically significant.

## Results

### MPTP administration increased the number of macrophages in the spleen, especially the classically activated M1 phenotype

**Figure 2:**
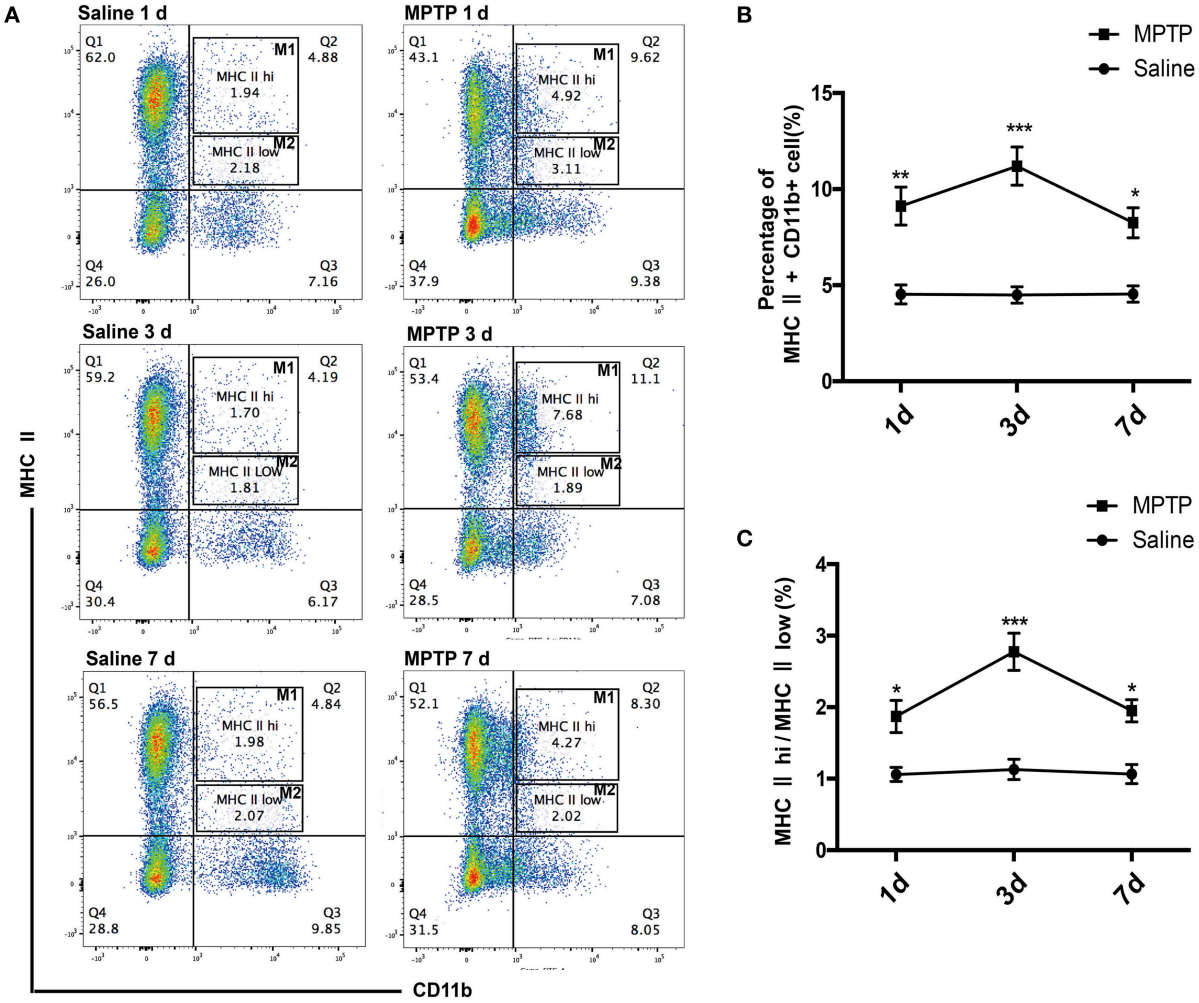
Peripheral activated macrophages, especially those of the classically activated M1 phenotype, are enriched in MPTP-induced PD mice. **(A)** Representative flow cytometry analysis of peripheral macrophage subpopulations in saline and MPTP mice. Macrophage subpopulations were characterized on the basis of MHC II (PE) and CD11b (FITC) staining. **(B)** Total number of macrophages in spleen as a percentage of total cells at different times. **(C)** The ratio of CD11b+ MHC II^hi^(M1) macrophages to CD11b+ MHC II^low^ (M2) macrophages at different times. Values are presented as the mean±SEM, ^*^p < 0.05, ^**^p < 0.01, ^***^p < 0.001 vs. saline treated group.

Macrophages are the pivotal regulatory cells of the peripheral immune system and play critical roles in the immune responses of all vertebrate species (Edholm et al., 2017). To assess the activation status of innate immune cells following MPTP injections, we used flow cytometry analysis to characterize subpopulations of splenic macrophages from PD mice and controls. We investigated the alteration of splenic macrophages at day 1, day 3 and one later time point at day 7 after MPTP injection. Our results revealed that the abundance of splenic macrophages was rapidly increased 1 day after MPTP administration in the PD group compared with the saline-treated group, as evidenced by an increase in the ratio of macrophages (CD11b+ MHC II+) (*t* = 5.864, *p* < 0.01; Figures 2A,B). This increase in splenic macrophages was observed until day 7. (3 days: *t* = 8.962, *p* < 0.001; 7 days: *t* = 4.626, *p* < 0.05; Figures 2A,B). Notably, the ratio of classically activated M1 macrophages (CD11b+ MHC II^hi^) to alternatively activated M2 macrophages (CD11b+ MHC II^low^) was increased after MPTP injection (1 days: *t* = 4.339, *p* < 0.05; 3 days: *t* = 10.089, *p* < 0.001;7 days: *t* = 4.895, *p* < 0.05; Figures 2A,C). The activation of peripheral macrophages probably plays an important role in the pathogenesis of PD.

### Depletion of peripheral macrophages alleviated MPTP-induced neurological deficits and neuronal death in the SNpc

**Figure 3:**
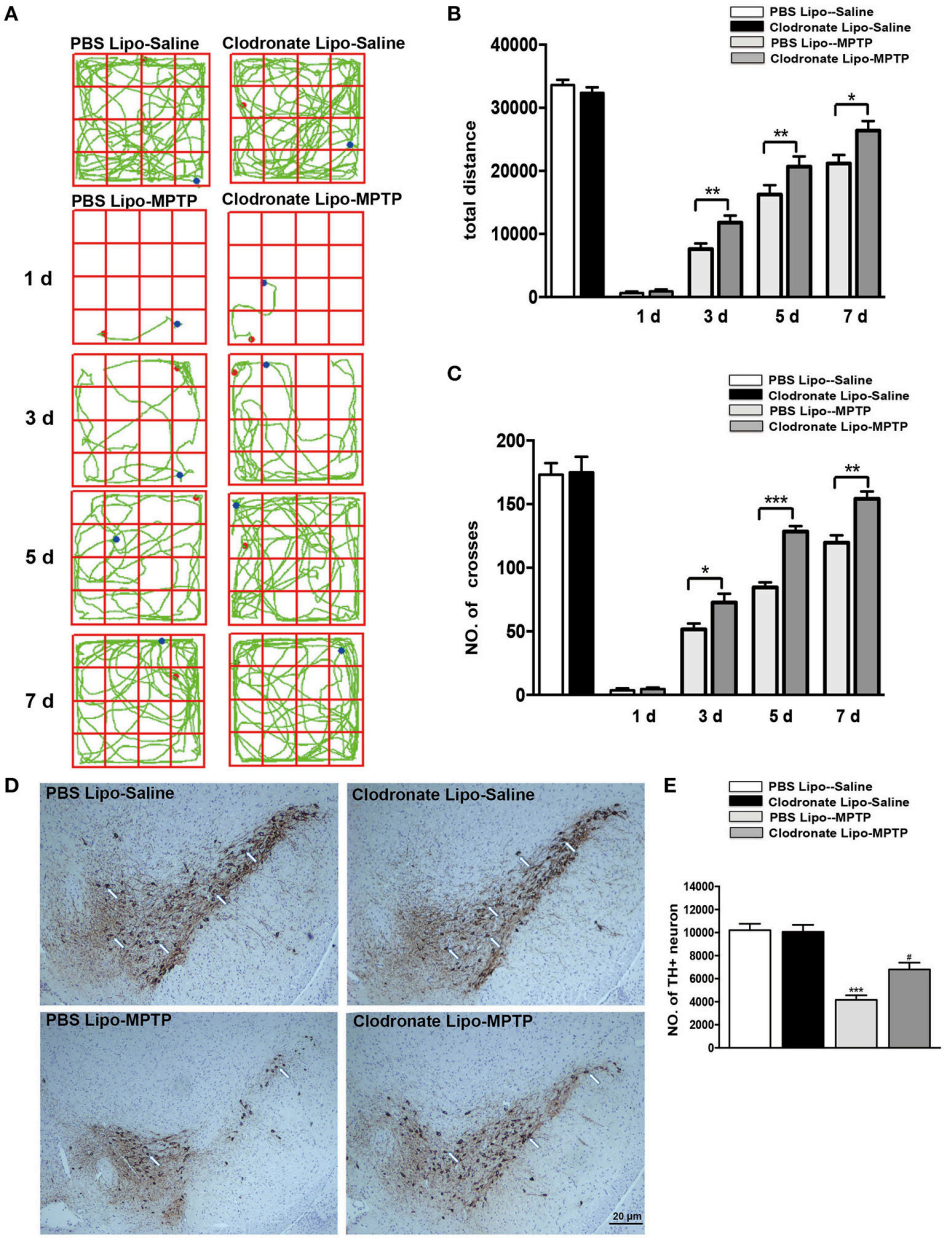
Clodronate liposome treatments improved behavioral deficits and reduced TH+ dopaminergic neuron loss in PD mice. **(A)** Movement paths in open-field test in different experimental groups. **(B)** Total distance traveled by different experimental groups. **(C)** Numbers of squares crossed by mice. ^*^p < 0.05, ^**^p < 0.01, ^***^p < 0.001 **(D)**. At 7 days after MPTP injection, loss of dopaminergic neurons was evaluated in the SNpc by immunohistochemical analysis of TH. Scale bar = 20 μm. **(E)** Quantification of TH+ cells in the SNpc in different experimental groups. Bars represent the mean total number of nigral TH+ dopaminergic neurons. ^***^p < 0.001, PBS Lipo-MPTP vs. PBS Lipo-saline. ^#^*p* < 0.05, Clodronate Lipo-MPTP vs. PBS Lipo-MPTP.

Activated macrophages probably play an important part in the pathogenesis of PD. To verify whether depletion of peripheral macrophages would influence the pathogenesis of PD, we used clodronate-encapsulated liposomes to cause irreversible depletion of macrophages *in vivo*, thereby efficiently decreasing the number of macrophages (Zeisberger et al., 2006). Mice that received clodronate or PBS liposomes showed no visible disorders such as infection, inhibition of motor activity. We first sought to determine whether depletion of macrophages affected MPTP-induced neurobehavioral deficits and dopaminergic neuronal death. For the open field test, we mapped the animals' paths on the 1st, 3rd, 5th, and 7th days, which reflected the total distance the mice traveled in 5 min. Saline-treated mice that received PBS liposomes or clodronate liposomes behaved normally. However, MPTP-treated mice that received PBS liposomes moved around the arena very little, preferring to stay still during the observation period, in contrast to MPTP-treated mice that received clodronate liposomes (3 days: *t* = 3.387, *p* < 0.01; 5 days: *t* = 3.939, *p* < 0.01; 7 days: *t* = 2.547, *p* < 0.05; Figures 3A,B). MPTP-treated mice also showed decreased locomotor activity, crossing fewer lines than saline-treated mice. MPTP-treated mice that received clodronate liposomes crossed more lines than MPTP-treated mice that received PBS liposomes (3 days: *t* = 2.546, *p* < 0.05; 5 days: *t* = 7.565, *p* < 0.001; 7 days: *t* = 4.232, *p* < 0.01; Figures 3A–C).

To determine whether depletion of peripheral macrophages in the MPTP model of PD is beneficial or harmful to neurons, we compared the clodronate liposome (lipo)-MPTP group with its PBS lipo-MPTP littermate group. We found that only 56% of the nigral TH+ neurons survived in the PBS lipo-MPTP group, while 76% of these neurons survived in the clodronate lipo-MPTP group (*F* = 30.36*, p* < 0.001; Figures 3D,E). These results suggested that depletion of peripheral macrophages protected against MPTP-induced brain injury, improved neurological outcome and reduced TH+ neuronal death in the SNpc.

### Clodronate liposome treatments mainly reduced the abundance of classically activated M1 macrophages

**Figure 4:**
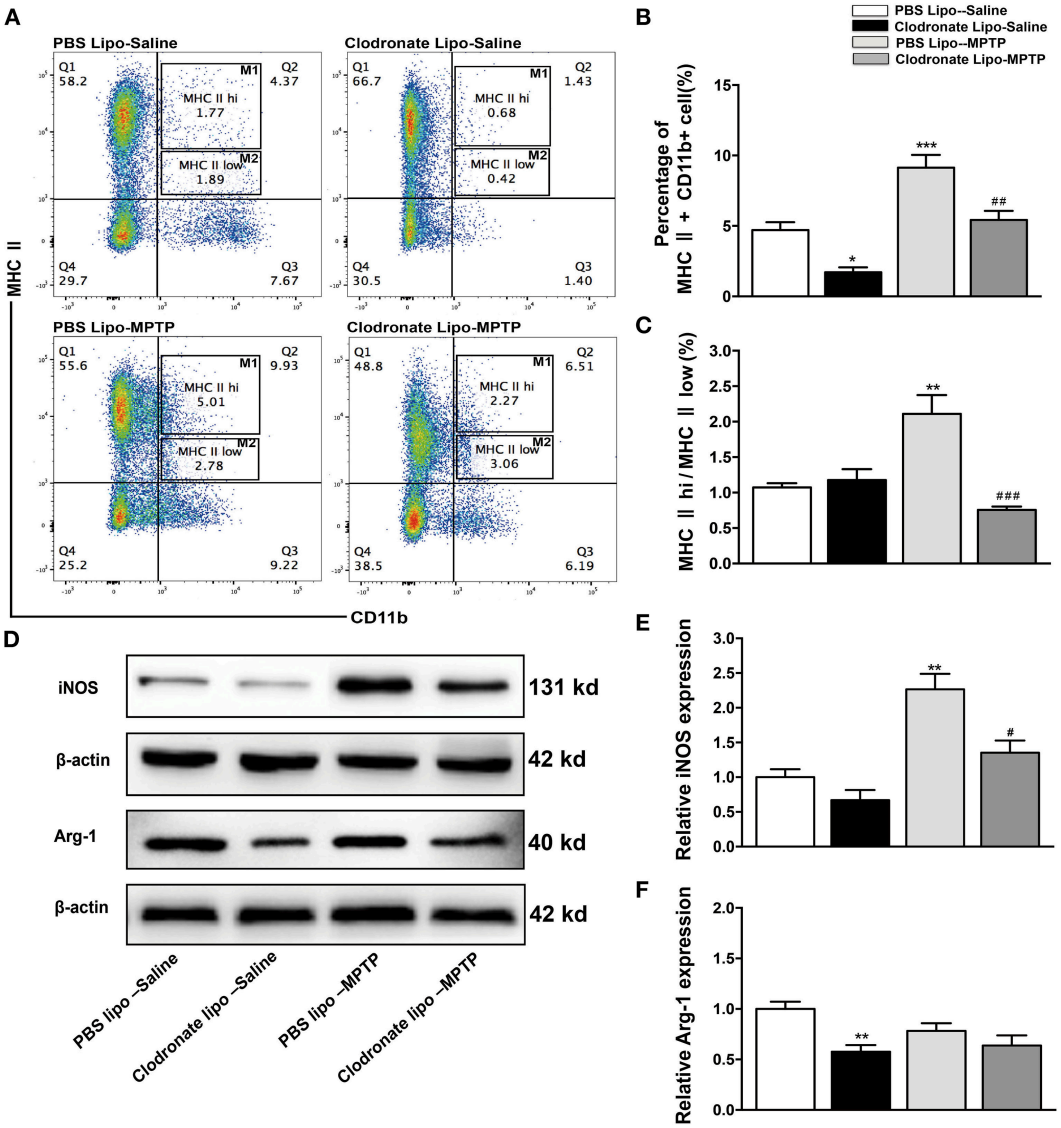
Clodronate liposome treatments successfully depleted peripheral macrophages in the spleen. **(A)** Flow cytometry analysis shows the percentage of MHC II+ CD11b+macrophages in the spleens of the PBS lipo-saline, clodronate lipo-saline, PBS lipo-MPTP, and clodronate lipo-MPTP groups at 7 days after MPTP injection. **(B)** Total splenic macrophage numbers as a percentage of total leukocytes in different experimental groups. **(C)** The ratio of CD11b+ MHC II^hi^ (M1) macrophages to CD11b+ MHC II^low^ (M2) macrophages in different experimental groups. **(D)** Analysis of iNOS and Arg-1 protein levels in the spleen at 7 days after MPTP injection. **(E)** Quantification of the densitometric value of the iNOS protein bands is shown, normalized to β-actin. **(F)** Quantification of the densitometric value of the Arg-1 protein bands, normalized to β-actin, is also shown. The gels were run under the same experimental conditions. Values are mean±SEM. ^*^p < 0.05, ^**^p < 0.01, ^***^p < 0.001 PBS Lipo-MPTP vs. PBS Lipo-saline. ^#^*p* < 0.05, ^##^*p* < 0.01, and ^###^*p* < 0.001 Clodronate Lipo-MPTP vs. PBS Lipo-MPTP.

Some researchers have reported that treatments with clodronate liposomes preferentially induce apoptosis of M1 monocytes/macrophages (Sunderkötter et al., 2004; Nahrendorf et al., 2007). Our study found that clodronate liposome treatment protected against MPTP-induced neurological deficits and neuronal death in the SNpc. Was the protective effect of clodronate liposomes associated with depletion of activated macrophages, especially depletion of classically activated M1 macrophages? To investigate whether the composition of peripheral macrophages differs between the clodronate-liposome-treated group and the PBS-liposome-treated group, we used flow cytometry to characterize splenic macrophage subpopulations at day 7 after the last MPTP injection. Our study showed that CD11b+ MHC II+ macrophages were depleted by 68.1% in the clodronate lipo-saline group compared with the PBS lipo-saline group and CD11b+ MHC class II+ macrophages were depleted by 42.9% in the clodronate liposome-MPTP mice compared to the PBS liposome-MPTP mice (*F* = 22.43, *p* < 0.001; Figures 4A,B). The ratio of the classically activated M1 phenotype (CD11b+ MHC II^hi^) to the alternatively activated M2 phenotype (CD11b+ MHC II^low^) was noticeably decreased in the clodronate lipo-MPTP group compared with the PBS lipo-MPTP group (*F* = 13.68, *p* < 0.001; Figures 4A,C).

As previously stated, the important hallmarks of M1 and M2 activation are iNOS and arginase activity, respectively (Edholm et al., 2017). Therefore, we used western blotting to analyze changes in the expression of M1 and M2 marker in the spleen. The results showed that MPTP could stimulate the expression of iNOS, an M1 polarization marker (*F* = 16.53, *p* < 0.01; Figures 4D,E), and slightly decrease the expression of Arg-1, an M2 polarization marker (*F* = 5.625, no statistical significance; Figures 4D,F) at day 7 in the PBS lipo-MPTP group compared with the PBS lipo-saline group. Clodronate liposomes reduced the expression of iNOS and Arg-1 (mainly iNOS) in lipo-MPTP mice compared with the PBS lipo-MPTP group (Figures 4D–F). We have provided the entire western blotting gels of inos and arg-1 expression in the Figure S1. Overall, our results demonstrated that clodronate liposomes can successfully deplete peripheral macrophages especially activated M1 phenotype in PD mice.

### The decrease in M1 macrophages inhibited activation of NF-κB signaling pathway and expression of MHC II

**Figure 5:**
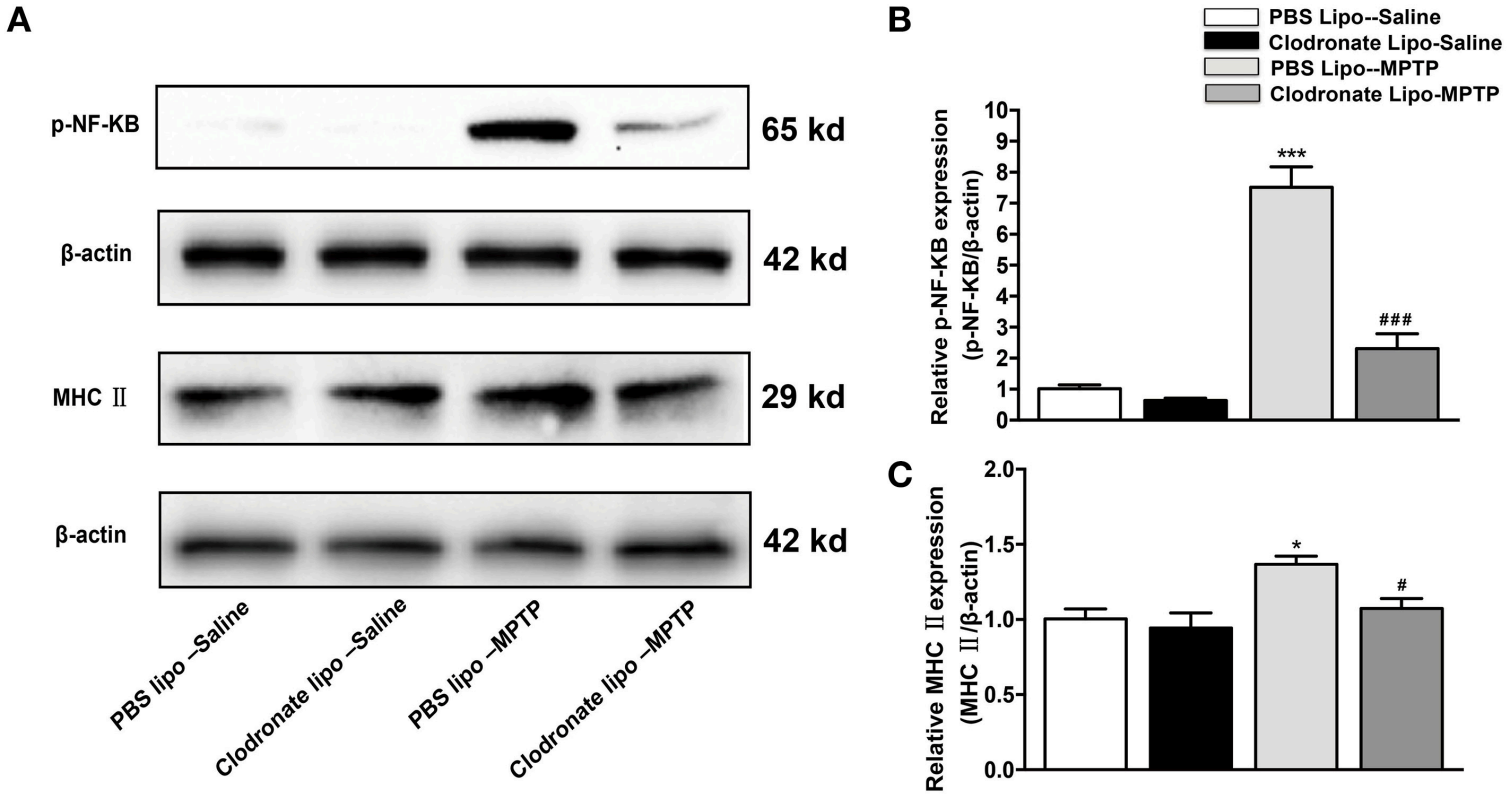
Clodronate liposome treatments reduced MPTP-induced phosphorylation of NF-κB and splenic expression of MHC II in PD mice. **(A)** Expression levels of p-NF-κB, and MHC II were analyzed by western blotting. **(B)** Quantification of the densitometric value of the p-NF-κB protein bands, normalized to β-actin, is shown. **(C)** Quantification of the densitometric value of the MHC II protein bands, normalized to β-actin, is shown. Values are presented as the mean±SEM from at least three independent experiments. ^*^p < 0.05, ^***^p < 0.001 PBS Lipo-MPTP vs. PBS Lipo-saline. ^#^*p* < 0.05, ^###^*p* < 0.001 Clodronate Lipo-MPTP vs. PBS Lipo-MPTP.

Studies have demonstrated that M1 macrophages are activated by pro-inflammatory stimuli via activation of NF-κB (Wu et al., 2016). We explored the NF-κB signaling pathway as a possible target for the protective effect of depletion of M1 macrophages in PD mice. In our study, we found that MPTP administration could induce increased phosphorylation of NF-κB in the PBS lipo-MPTP group compared with the PBS lipo-saline group. Nevertheless, clodronate liposomes partially reduced the MPTP-induced phosphorylation of NF-κB in the clodronate lipo-MPTP group (*F* = 59.42, *p* < 0.001; Figures 5A,B). The expression of MHC II is restricted to antigen-presenting cells (APCs), which are responsible for the presentation of peptide antigens to T cells. We investigated the expression change of MHC II in the spleen under different treatments. We detected that the expression of MHC II increased in the PBS lipo-MPTP group compared with the PBS lipo-saline group (*F* = 4.701, *p* < 0.05; Figures 5A,C). Clodronate liposomes decreased MHC II expression in the clodronate lipo-MPTP group compared with the PBS lipo-MPTP group (*F* = 4.701, *p* < 0.05; Figures 5A,C). We have provided the entire western blotting gels of p-NF-κB and MHC II expression in the Figure S1.

### Infiltration of T cells into the brain was reduced after M1 macrophage depletion in the PD model

**Figure 6:**
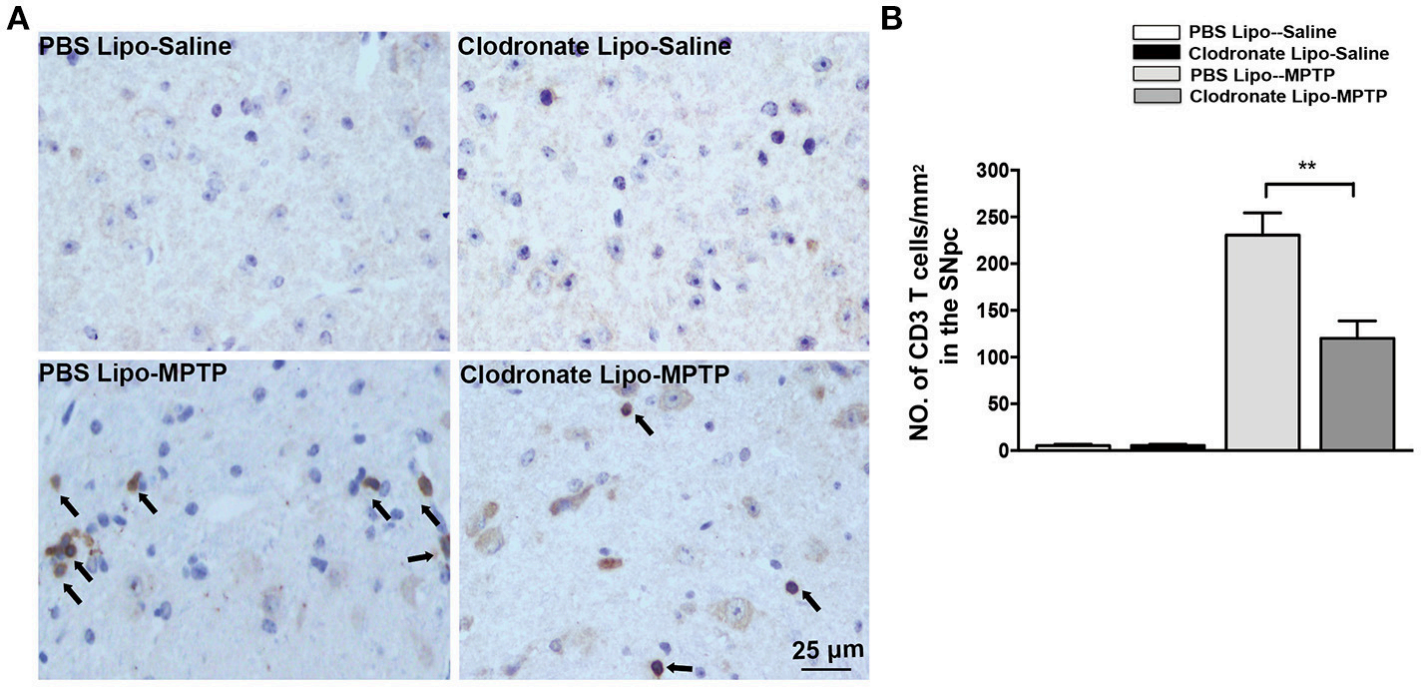
Clodronate liposome treatment reduced infiltration of T cells into the brain. **(A)** Brain slices were stained for the T cells marker CD3 as shown in pictures of the midbrain. Scale bar = 25 μm. **(B)** Quantification of the total number of infiltrated CD3+ T cells in the SNpc from different experimental groups. Values are presented as the mean±SEM. ^**^p < 0.01, Clodronate Lipo-MPTP vs. PBS Lipo-MPTP.

Emerging evidence has demonstrated that T cells infiltrate into the substantia nigra in PD patients and in animal models of PD (González et al., 2013). M1 macrophage depletion reduced MHC II expression, which may prevent antigen presentation to T cells. To examine the effect of M1 macrophage depletion on T cell infiltration into the brain, we detected CD3+ T cells using immunohistochemistry. We found a marked reduction in CNS-infiltrating T cells in the SNpc in the clodronate lipo-MPTP group compared with the PBS lipo-MPTP group (*F* = 51.07, *p* < 0.001; Figures 6A,B). This result showed that depletion of classically activated M1 macrophages could decrease the invasion of T cells into the brain.

### Inflammatory cytokine expression and microglial activation were inhibited after M1 macrophage depletion in the striatum and SNpc

**Figure 7:**
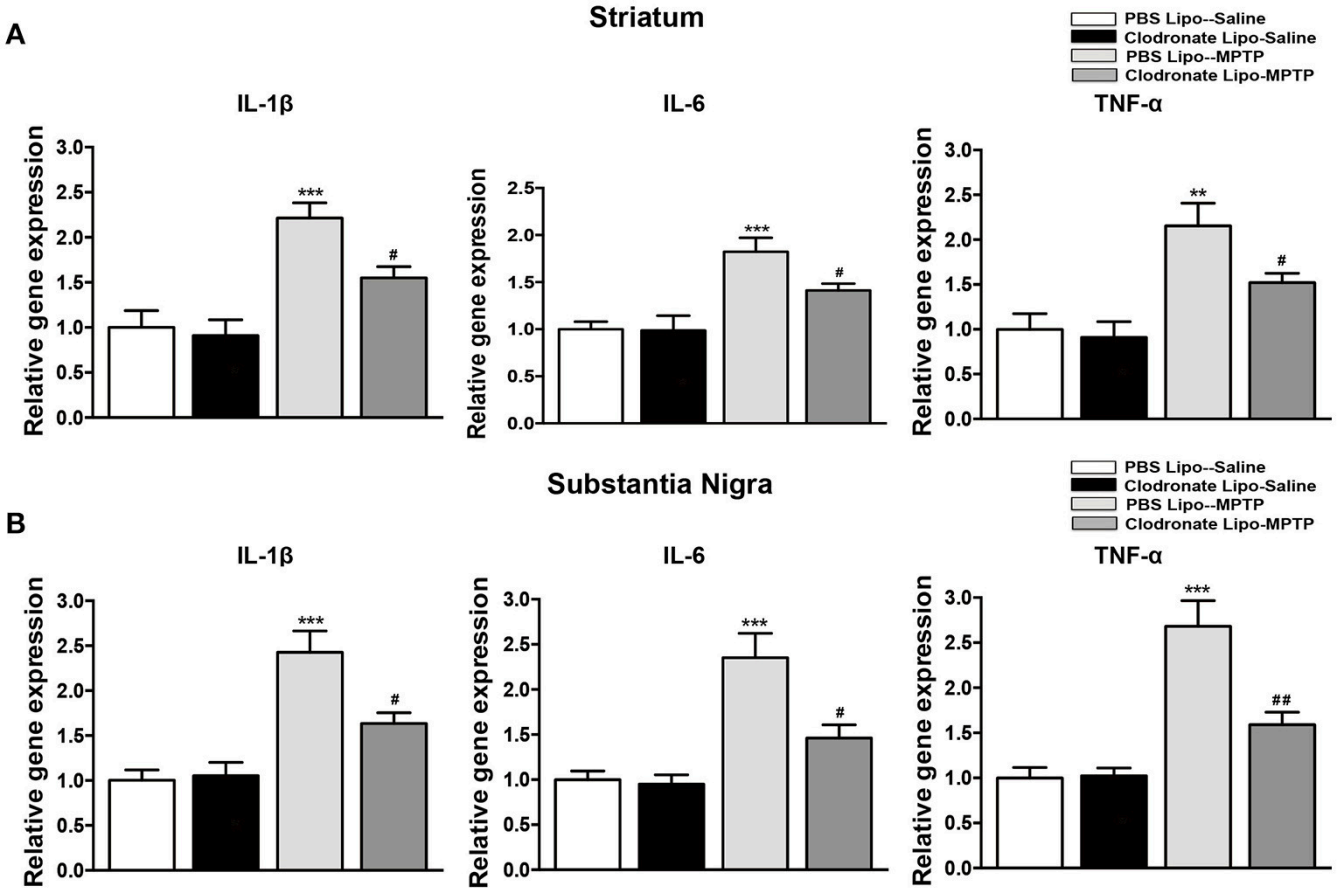
Clodronate liposome treatment reduced inflammatory cytokine expression in the striatum and SNpc. **(A)** mRNA levels of the pro-inflammatory mediators IL-1β(a), IL-6(b), and TNF-α(c) normalized to Rplp0 in the striatum. **(B)** mRNA levels of the pro-inflammatory mediators IL-1β(a), IL-6(b), and TNF-α(c) normalized to Rplp0 in the SN. Data are mean ± SEM, *n* = 6 per group. ^**^p < 0.01, ^***^p < 0.001 PBS Lipo-MPTP vs. PBS Lipo-saline. ^#^*p* < 0.05, ^##^*p* < 0.01, Clodronate Lipo-MPTP vs. PBS Lipo-MPTP.

The infiltration of activated T cells into the brain releases inflammatory cytokines to further activate microglia (Depboylu et al., 2012), and the T cells are also restimulated by resident antigens presented by microglia (Bogie et al., 2014). We assessed the effect of M1 macrophage depletion on inflammatory cytokine expression and microglial activation. Mice in the PBS lipo-MPTP group revealed a prominent increase in the release of the inflammatory cytokines IL-1β, IL-6, and TNF-a in the striatum (*F* = 14.03, *p* < 0.001, Figure 7A a; *F* = 10.43, *p* < 0.001, Figure 7A b; TNF-a, *F* = 9.699, *p* < 0.001, Figure 7A c) and SNpc (*F* = 16.64, *p* < 0.001, Figure 7B a; IL-6*, F* = 12.89, *p* < 0.001, Figure 7B b; *F* = 20.49, *p* < 0.001, Figure 7B c) compared with the PBS lipo-saline group. Depletion of M1 macrophages partially prevented the MPTP-induced release of inflammatory cytokines in the clodronate lipo-MPTP group (Figures 7A,B).

**Figure 8:**
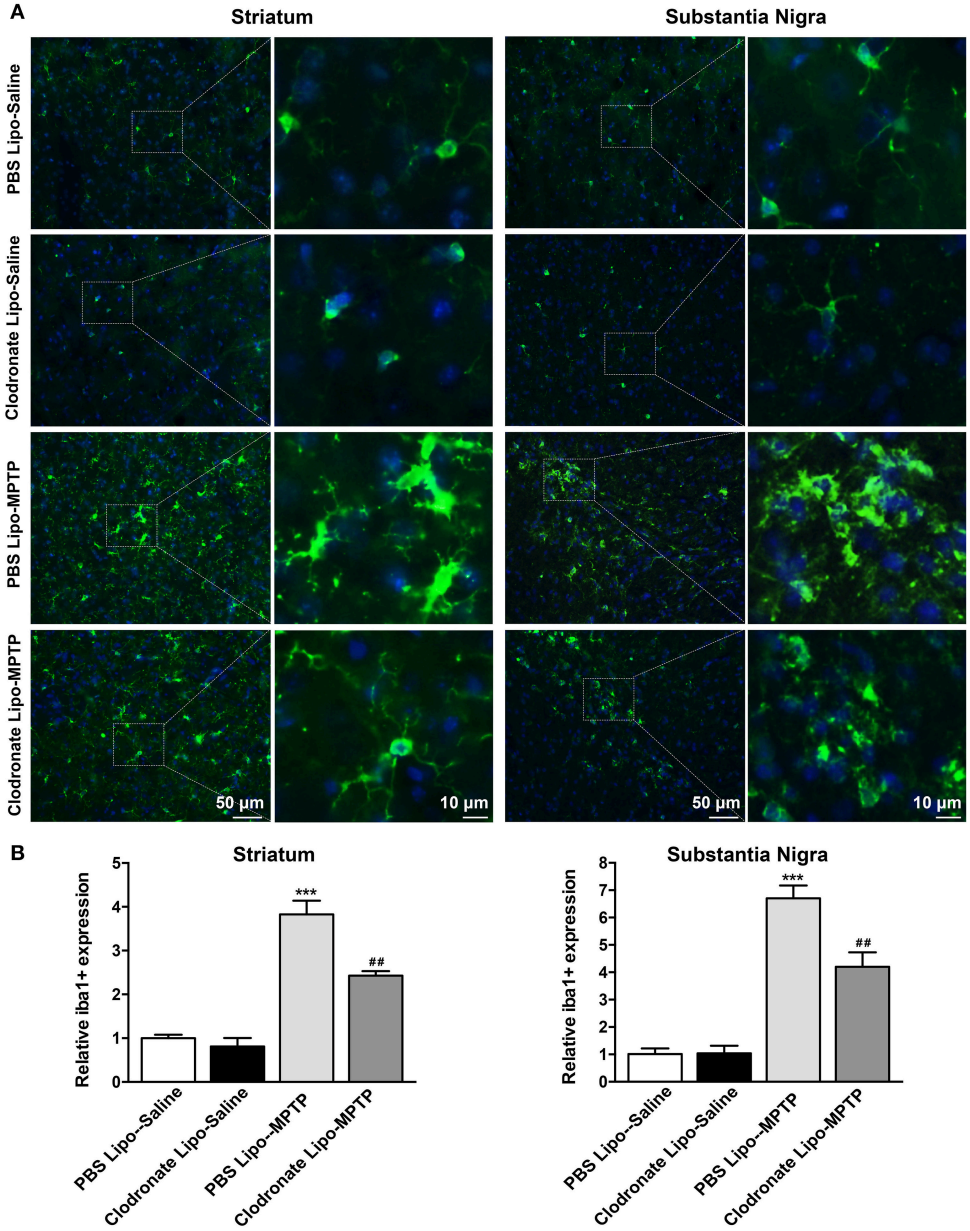
Clodronate liposome treatment inhibited microglial activation in the striatum and SNpc. **(A)** Microglial activation was determined in the striatum(a) and SN(b) by immunohistochemical analysis of Iba1. Photomicrographs show Iba1 (green)-stained microglia in the striatum and SN at 7 days in PBS lipo-saline, clodronate lipo-saline, PBS lipo-MPTP, and clodronate lipo-MPTP mice. The right panels by each column present a higher magnification of the images in the left panels labeled by the boxes. Scale bar = 50 μm in left panels and 10 μm in right panels. **(B)** The quantity of microglia in the striatum(a) and SN(b) was quantified by the intensity of Iba-1+ immunofluorescence. Data were normalized against the Iba1+ level of the PBS-lipo saline group. Data are mean±SEM, *n* = 4 per group. ^***^p < 0.001 PBS Lipo-MPTP vs. PBS Lipo-saline. ^##^*p* < 0.01 Clodronate Lipo-MPTP vs. PBS Lipo-MPTP.

Meanwhile, the results showed that MPTP could activate microglia in the striatum (*F* = 53.14, *p* < 0.001; Figure 8B a) and SNpc (*F* = 54.26, *p* < 0.001; Figure 8B b) in PBS lipo-MPTP group compared with the PBS lipo-saline group. Depletion of M1 macrophages inhibited the activation of microglia in the clodronate lipo-MPTP group compared with the PBS lipo-MPTP group (Figures 8A,B). These results demonstrated that the depletion of M1 macrophages could reduce inflammatory cytokine expression and ameliorate microglial activation in the striatum and SNpc of PD mice.

## Discussion

In our study, we demonstrated that the number of M1 macrophages in the peripheral immune system was increased in an MPTP PD model. Depletion of M1 macrophages by clodronate liposomes reversed these inflammatory effects and blunted the loss of TH+ nigral neutrons and functional impairments caused by MPTP.

Under normal physiological conditions, peripheral immune cells dynamically survey the surrounding environment and have ability to inflammation-suppressing. Under pathological conditions, over-activated immune cells migrate toward the lesion site, produce neurotoxic factors such as pro-inflammatory cytokines. Recent research showed that inflammatory cytokines in peripheral immune system might amplify neuroinflammation and contribute to the pathogenesis of PD (Perry et al., 2007; Cunningham et al., 2009; Ferrari and Tarelli, 2011). Macrophages are the crucial regulatory cells of the peripheral immune system. Therefore, we first analyzed the peripheral macrophage subpopulations of spleen in PD mice and controls. Our results revealed that MPTP administration increased the number of macrophages in the spleen. This indicate that the increase and activation of peripheral macrophages are probably involved in the pathogenesis of PD.

Recently, more and more evidences reveal that immune system involvement in PD susceptibility, specifically in the context of M1 and M2 macrophages activation states. Polarized macrophages are mainly subdivided into classically activated M1 phenotype and alternatively activated M2 phenotype. Our research found that M1 macrophages and M2 macrophages were both increased at 1 day after MPTP injection. As the disease continues to advance, the ratio of M1 macrophages to M2 macrophages was increased until day 7 after the last MPTP injection. To study the role of activated macrophages in the pathogenesis of PD, we used intraperitoneal injection of clodronate liposomes to deplete the macrophages in the periphery. Clodronate liposomes are widely used to deplete peripheral macrophages (Gliem et al., 2012; Godwin et al., 2013). Our study demonstrated that reducing the population of peripheral macrophages protected against MPTP-induced neurological deficits and neuron reduction in the SNpc.

In the next part of this study, we counted splenic macrophages according to their immunophenotypes in saline-treated mice and MPTP-treated mice that received PBS liposome and clodronate liposome treatments. Our study found that M1 macrophages were mainly depleted compared with M2 macrophages in the mice of the clodronate lipo-MPTP group. The results of western blotting also showed that clodronate liposome mainly inhibited the expression of iNOS, a M1 polarization marker, and had little influence on the expression of Arg-1, a M2 polarization marker in MPTP-treated mice. This is because inflammatory M1 macrophages are more capable of phagocytosing clodronate liposomes. It has also been established that clodronate encapsulated liposomes efficiently induce apoptosis of M1 macrophages (Sunderkötter et al., 2004; Nahrendorf et al., 2007). M1 macrophages release more pro-inflammatory cytokines by activating the NF-κB signaling pathway (Yang et al., 2016) and have greater antigen-presenting capacity through expression of MHC II (Gordon, 2003). Our study showed that clodronate liposomes could inhibit the MPTP-induced phosphorylation of NF-κB and reduce the expression of MHC II in the spleen.

The activation of the NF-κB signaling pathway and increase in the expression of MHC II could activate T cells to infiltrate into the brain (Cho et al., 2014; Yang et al., 2016). Our study demonstrated that MPTP administration promoted T cells infiltration into the brain. M1 macrophage depletion reduced the infiltration of activated T cells into the brain. In addition to studying T cells, we also measured the release of inflammatory cytokines and the activation of microglia in the striatum and SNpc. Microglia are the first-line of defense in the brain in case of infectious or pathological events. The activation of microglia can induce detrimental neurotoxic effects by excessive production of inflammatory cytokines (Murray et al., 2014). Our results showed that decrease in M1 macrophages could reduce inflammatory cytokine release and ameliorate microglial activation in the striatum and SNpc of PD mice.

**Figure 9:**
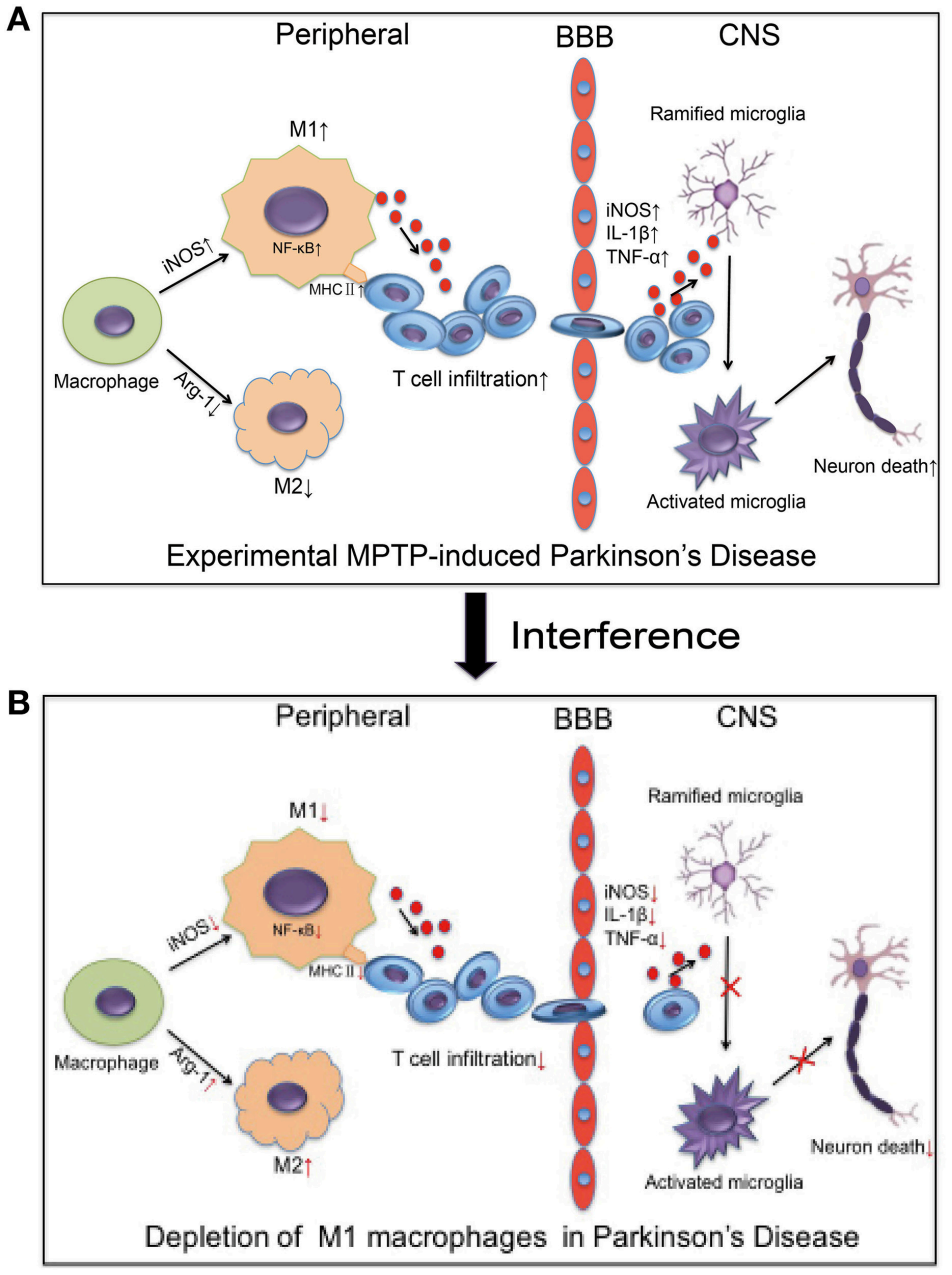
Relationship between macrophage polarization and neuronal damage in the PD mouse brain and peripheral immune system. **(A)** The figure illustrates that experimental MPTP-induced PD can increase M1 macrophages and decrease M2 macrophages in peripheral system. Furthermore, it promoted the infiltration of T cells into the brain, which increase the release of inflammatory cytokines and eventually cause neuronal death. **(B)** The demonstrated depletion of M1 macrophages reduced the infiltration of activated T cells into the brain and ameliorated the neuroinflammatory events and neuronal cell death in the MPTP-induced PD mice model.

In summary, our study demonstrated that MPTP administration increased the number of M1 macrophages. The increasing M1 macrophages activated the NF-κB signaling pathway and increased the expression of MHC II. This process may promote the infiltration of activated T cells into the brain, which induced the death of dopaminergic neurons in the brain (Figure 9A). Depletion of M1 macrophages reduced the infiltration of activated T cells into the brain, thereby ameliorating neuroinflammation and dopaminergic neuronal cell death in MPTP-induced PD mouse model (Figure 9B). The inhibition of M1 macrophages could be evaluated as a potential therapeutic approach for PD.

## Ethics statement

All animals survived under Specific Pathogen Free (SPF) environment in a laboratory within the animal facility located at Xinhua Hospital affiliated to Shanghai JiaoTong University, School of Medicine. All mice were housed under standardized. Laboratory conditions and monitored to observe changes in ordinary conditions and activities. Animal care and use were in Accordance with the guidelines established by the Administration of Affair Concerning Laboratory Animals for Shanghai JiaoTong University, the National Institutes of Health Guide for care and Use of Laboratory Animals (GB14925-2010) and the Regulations for the Administration of Affairs Concerning Experimental Animals (China, 2014).

## Author contributions

ZL and AY: designed experiments; AY, YZ, JL, LS, and XW: performed the experiments; AY and YZ: analyzed the results; AY: wrote the manuscript with contributions from ZL. All authors read and approved the final manuscript.

## Conflict of interest statement

The authors declare that the research was conducted in the absence of any commercial or financial relationships that could be construed as a potential conflict of interest.

## Supplementary material

Figure S1 The entire western blotting gels of iNOS, Arg-1, p-NF-κB, MHC II expression.

Supplementary Video 1 PBS Lipo-Saline group.

Supplementary Video 2 Clodronate Lipo-Saline group.

Supplementary Video 3 1 d PBS Lipo-MPTP group.

Supplementary Video 4 1 d Clodronate Lipo-MPTP group.

Supplementary Video 5 3 d PBS Lipo-MPTP group.

Supplementary Video 6 3 d Clodronate Lipo-MPTP group.

Supplementary Video 7 5 d PBS Lipo-MPTP group.

Supplementary Video 8 5 d Clodronate Lipo-MPTP group.

Supplementary Video 9 7 d PBS Lipo-MPTP group.

Supplementary Video 10 7 d Clodronate Lipo-MPTP group.
