# Supplementary figures and images for: Partial Depletion of Peripheral M1 Macrophages Reverses Motor Deficits in MPTP-Treated Mouse by Suppressing Neuroinflammation and Dopaminergic Neurodegeneration
Source: Front Aging Neurosci. 2018 Jun 5;10:160. doi: 10.3389/fnagi.2018.00160 (PMC5996129; doi:10.3389/fnagi.2018.00160)

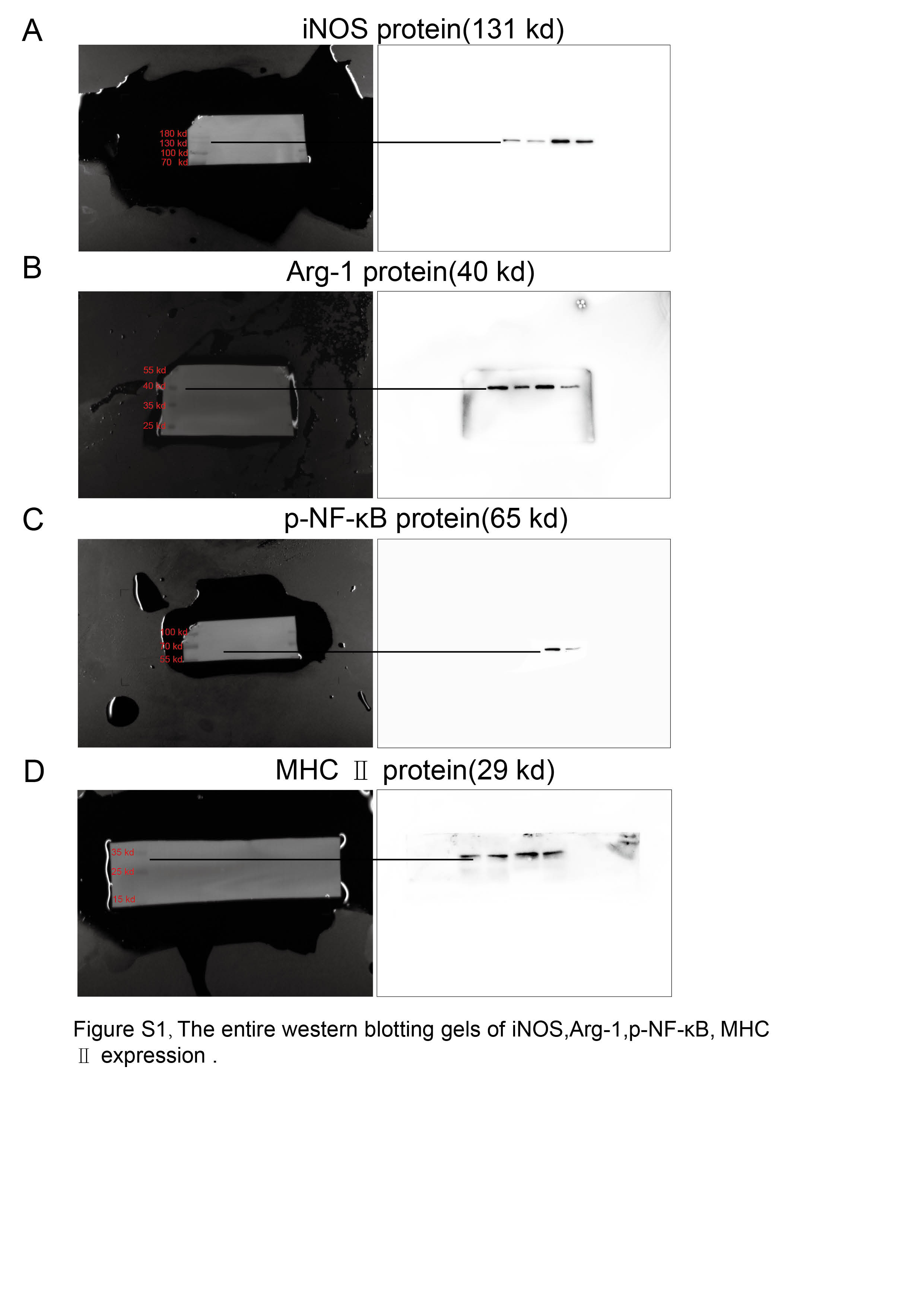

Supplement: Figure S1 — The entire western blotting gels of iNOS, Arg-1, p-NF-κB, MHC II expression. [file Image_1.JPEG]
